# Evaluation of the Neuroprotective Potential of *Sutherlandia frutescens* in a Rotenone-Induced Rat Model of Parkinson's Disease

**DOI:** 10.1155/bn/6606560

**Published:** 2025-03-11

**Authors:** Lilit Darbinyan, Karen Simonyan, Larisa Manukyan, Vaghinak Sarkisian, Lusya Hovhannisyan, Lilia Hambardzumyan

**Affiliations:** ^1^Sensorimotor Integration Lab, Orbeli Institute of Physiology NAS RA, Yerevan, Armenia; ^2^Neuroendocrine Relationships Lab, Orbeli Institute of Physiology NAS RA, Yerevan, Armenia; ^3^G.S. Davtyan Institute of Hydroponics Problems NAS RA, Yerevan, Armenia

**Keywords:** in vivo electrophysiology, open-field test, Parkinson's disease, *Sutherlandia frutescens*

## Abstract

*Sutherlandia frutescens* (*SF*) is a plant used traditionally in South Africa for various health conditions, including neurological disorders. Parkinson's disease (PD) is a progressive neurodegenerative disorder characterized by the degeneration of dopaminergic neurons in the substantia nigra, resulting in motor symptoms. Rotenone, a pesticide, has been linked to PD-like symptoms in both in vitro and in vivo studies. However, *SF*-specific effects of *SF* on PD-related symptoms have not been extensively studied. This study was aimed at investigating the potential neuroprotective effects of *SF* against rotenone-induced PD using in vivo electrophysiological recordings from the hippocampus and an open-field test to assess motor behavior. Rats were divided into three groups: a control group receiving sunflower oil, a rotenone group treated with rotenone (2.0 mg/kg), and an *SF* group treated with hydroponically grown *SF* extract. Electrophysiological recordings from the hippocampus were conducted to assess neuronal activity, and an open-field test was used to evaluate motor behavior. Rats treated with *SF* exhibited significantly higher motor activity compared to both the sunflower oil and rotenone groups, suggesting an activating effect of *SF* on motor behavior. In contrast, the rotenone group displayed reduced activity levels and exploratory behavior, highlighting the suppressive impact of rotenone on motor function. These findings suggest that *SF* modulates hippocampal neuronal activity and may offer neuroprotective benefits against rotenone-induced PD-like symptoms. *SF*, a plant with traditional medicinal applications, shows potential in modulating motor behavior and hippocampal neuronal activity in a rotenone-induced PD model. Further studies are needed to clarify the underlying mechanisms and evaluate the clinical relevance of *SF* in PD management.

## 1. Introduction

*Sutherlandia frutescens* (L.) (*SF*), also taxonomically referred to as *Lessertia frutescens*, belongs to the legume family (Fabaceae). It has been used in traditional medicine for centuries by various cultural groups in southern Africa to manage a wide range of ailments, including gynecological, gastrointestinal, and urogenital disorders. Notably, the review mentions that no adverse treatment outcomes have been recorded from its traditional use [1]. Several studies have investigated the anticancer properties of *SF*. In vitro studies have demonstrated the antiproliferative effects of *SF* extracts on cancer cells and diabetes [2, 3]. A few case reports suggest *SF* may help decrease fatigue in cancer patients, but its efficacy as a cancer treatment has not been conclusively proven in human studies [4]. Phytochemical evaluations of this plant have shown that it contains significant amounts of pharmacologically important constituents, including gamma-aminobutyric acid, glycosides, saponins, and L-canavanine [5]. *SF*, a plant with medicinal properties, has been studied for its potential effects on neurodegenerative disorders (NDDs). Ethanol extracts of *SF* have been shown to suppress NMDA-induced reactive oxygen species (ROS) production in neurons, which suggests that *SF* may have neuroprotective effects against oxidative stress [6]. *SF* is listed among herbal drugs used for the treatment of NDDs, which include Alzheimer's disease, Parkinson's disease (PD), and amyotrophic lateral sclerosis [7]. Additionally, studies have demonstrated the neuroprotective effect of *SF* extracts against oxidative and inflammatory responses, as well as the anti-inflammatory activities of the plant [8, 9]. *SF* consumption was found to mitigate microglial activation in the hippocampus and striatum of ischemic brains of mice [10]. Despite its long history of traditional use and promising research findings, more clinical studies are needed to fully understand the safety and efficacy of *SF* in treating various conditions. Nonetheless, the available evidence suggests that *SF* is a plant with significant medicinal potential that warrants further investigation.

PD is a neurodegenerative disease associated with aging, characterized by tremor, bradykinesia, rigidity, and postural instability. It is the second most common neurodegenerative disease after Alzheimer's and currently affects 1.5% of the world's population over 65 years of age. The pathological hallmark of PD is the loss of dopaminergic neurons in the substantia nigra, leading to a deficit of dopamine in the striatum [11, 12]. PD is associated with hippocampal changes, although the hippocampus is not part of the nigrostriatal dopaminergic pathway.

The rotenone-induced rat model is a well-established and widely used animal model for studying PD, where chronic exposure to rotenone, a mitochondrial complex I inhibitor, leads to key features of PD in rats, including the loss of dopaminergic neurons in the substantia nigra, motor impairments such as deficits in rotarod and hanging wire tests, abnormal neuronal activity in the subthalamic nucleus (STN) characterized by a higher discharge rate, more bursts per minute, and increased oscillatory activity, as well as increased relative beta power in the STN and motor cortex [13].

Hippocampal atrophy has been observed in PD patients compared to controls, and this atrophy is related to cognitive impairment, particularly memory deficits [14, 15]. The distribution of *α*-synuclein in the hippocampus is not associated with volumetric changes, but proteomic analysis reveals alterations in synaptic proteins, suggesting that hippocampal changes occur at the synapse level during PD [16]. Hippocampal functional connectivity changes have also been observed in PD patients, with reduced connectivity to the paracingulate gyri [17].

The hippocampus's involvement in PD is increasingly recognized, particularly in relation to nonmotor symptoms such as cognitive deficits, anxiety, and depression. These symptoms are significant contributors to the disease burden and often precede motor symptoms by years. Research has demonstrated hippocampal atrophy, altered functional connectivity, and synaptic protein changes in PD patients, highlighting the vulnerability of this brain region in the disease process [18–20].

The hippocampus is integral to memory formation, emotional regulation, and exploratory behavior. Dysfunction in this region has been linked to reduced exploratory behavior and impaired adaptation to novel environments in rodent models—behaviors that overlap with PD-related deficits. Additionally, neuropsychiatric manifestations such as depression and anxiety, which are more debilitating than motor symptoms for many patients, often involve hippocampal dysfunction [21, 22]. In the context of your study, the improved exploratory behavior observed in the RSF group during the open-field test may correlate with hippocampal involvement. *SF* treatment could influence hippocampal synaptic activity and plasticity, potentially modulating both nonmotor and motor-related symptoms of PD. Emphasizing this link in your manuscript strengthens the argument for targeting hippocampal mechanisms as part of a broader therapeutic strategy for PD symptomatology [18, 23].

Although *SF* has been reported to show activity in various disease models, studies on its neuroprotective activity in a PD model have not been reported. This study therefore investigated the mechanisms by which *SF* could potentially provide neuroprotection against rotenone-induced rat model of PD.

## 2. Results

This study investigated the neuroprotective effects of *SF* in a rotenone-induced rat model of PD.

The measurement parameters are as follows:

1. Electrophysiological recordings: Extracellular hippocampal spike activity was recorded after high-frequency stimulation (HFS) of the entorhinal cortex (EC) to assess synaptic plasticity.
2. Open-field test (OFT): Behavioral assessments included total distance traveled and line crossings to evaluate locomotor activity and exploratory behavior.

### 2.1. In Vivo Extracellular Recordings

**Figure 1:**
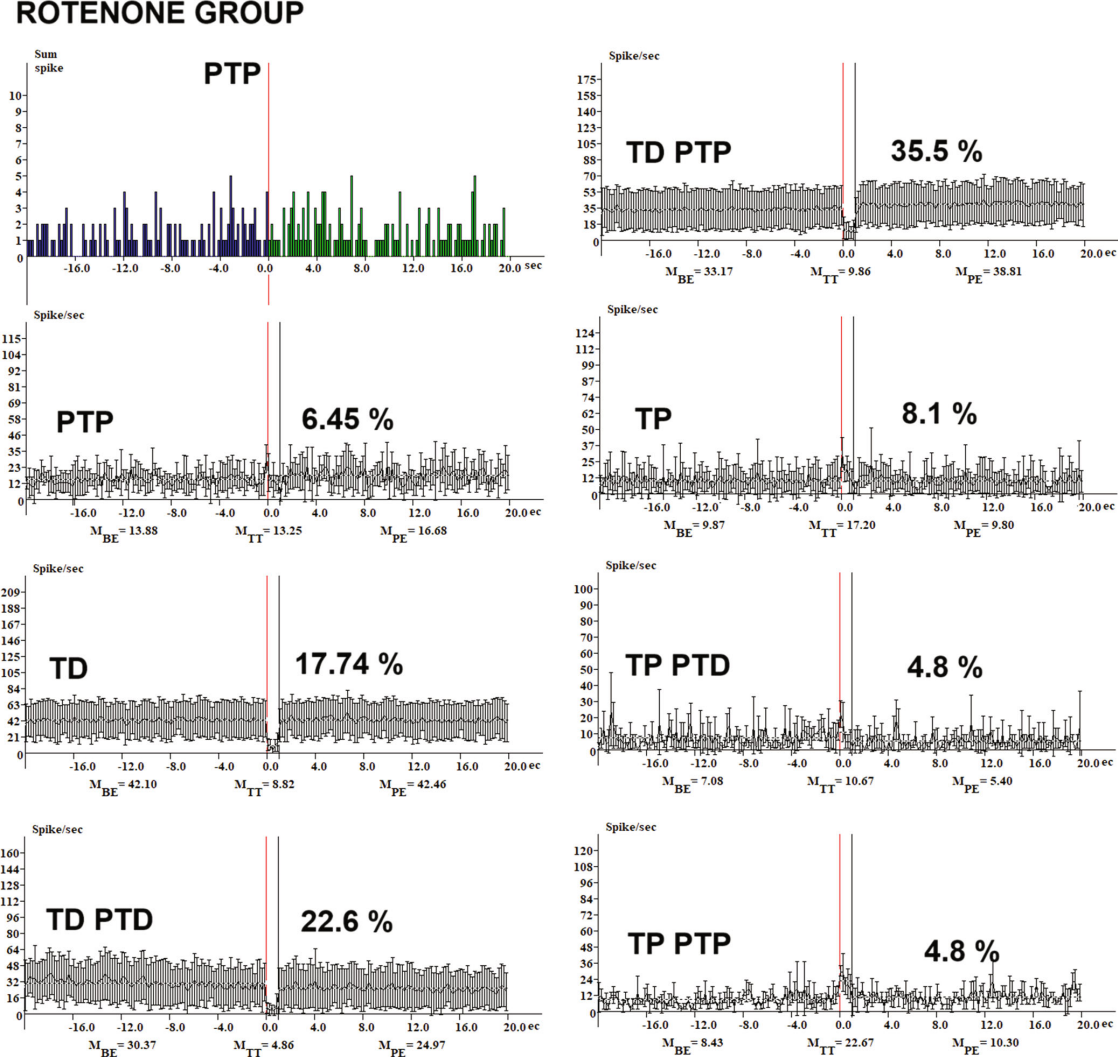
Histograms of peristimulus frequency of spike activity and the percentage distribution of response types in hippocampal neurons following HFS of the EC in the rotenone (R) group are presented. Real-time spike distribution is shown: BE, PE, and during HFS (TT).

**Figure 2:**
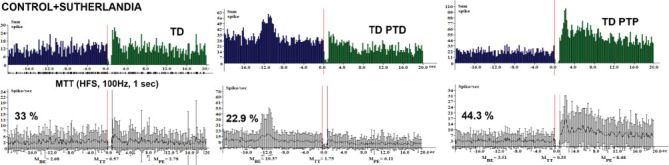
Histograms showing peristimulus spike activity frequency and the percentage distribution of response types in hippocampal neurons in the control + *Sutherlandia* (CSF) group following high-frequency stimulation (HFS) of the entorhinal cortex (EC). Spike frequencies are presented for three intervals: before stimulation (BE, 20 s), during HFS (TT, 1 s at 100 Hz), and poststimulation (PE, 20 s). Mean spike frequencies are labeled as MBE, MTT, and MPE, respectively. The blue line represents the amplitude discriminator used for spike selection. The percentage distribution of excitatory and inhibitory hippocampal neuronal responses is illustrated, reflecting diverse activity patterns in response to HFS.

**Figure 3:**
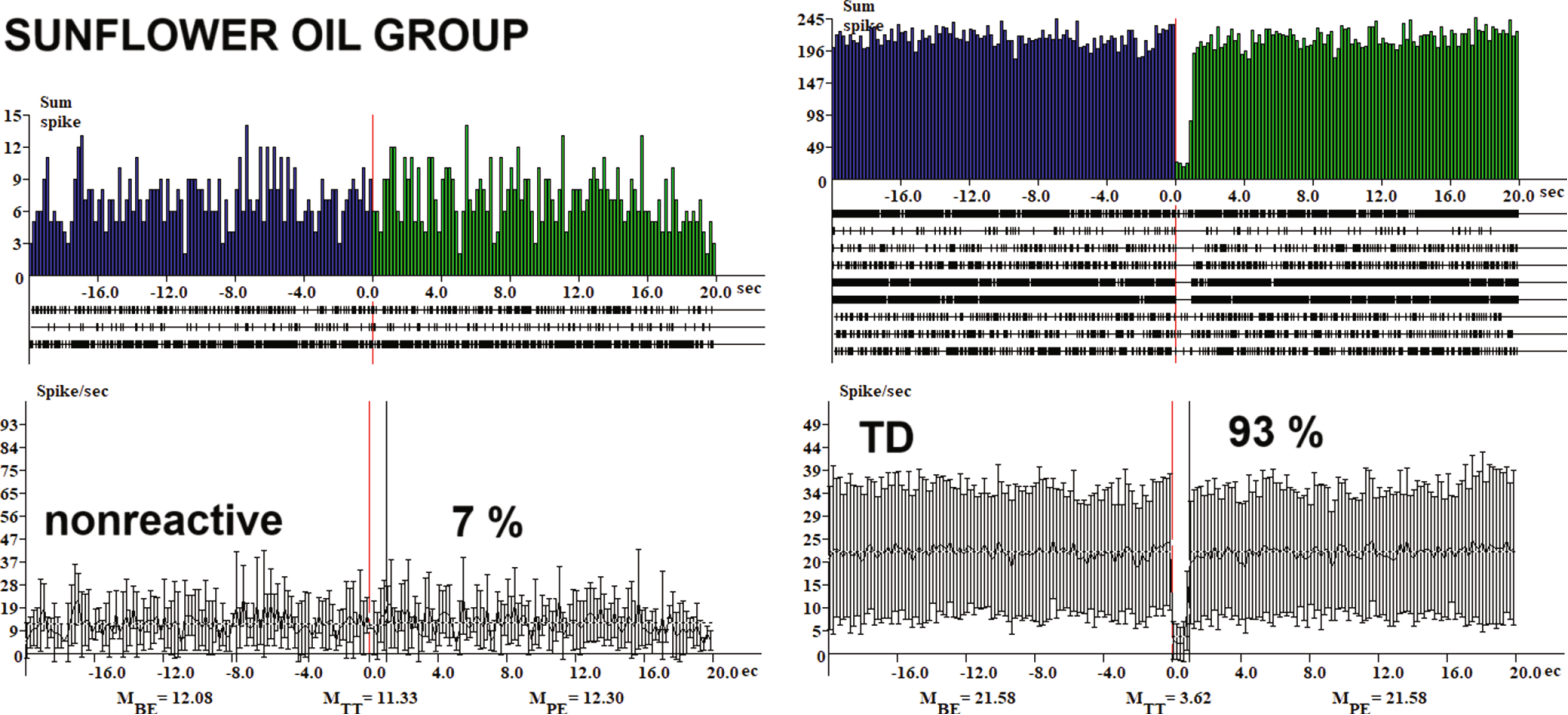
Histograms of peristimulus frequency of spike activity and the percentage distribution of response types in hippocampal neurons following HFS of the EC in the sunflower oil (SO) group are presented. Real-time spike distribution is shown: BE, PE, and during HFS (TT).

The analysis of neuronal responses across various treatment groups revealed significant differences in the percentages of tetanic depression (TD) neurons and related phenomena. Conversely, in the rotenone group (Figure 1), the percentage of TD neurons decreased markedly to 17.74%. Additionally, tetanic potentiation and post-tetanic depression (TP-PTD) and tetanic potentiation and post-tetanic potentiation (TP-PTP) were observed at 4.8% each. Post-tetanic potentiation (PTP) was recorded at 6.45%, while TD-PTD and TD-PTP were noted at 22.6% and 35.5%, respectively. In the control + *Sutherlandia* group (Figure 2), TD neurons accounted for 33%, while the combination of tetanic depression and post-tetanic depression (TD-PTD) was 22.95%, and tetanic depression and post-tetanic potentiation (TD-PTP) reached 44.3%. In the sunflower oil-treated group (Figure 3), 93% of neurons exhibited TD, with the remaining 7% classified as nonreactive (Figure 3).

**Figure 4:**
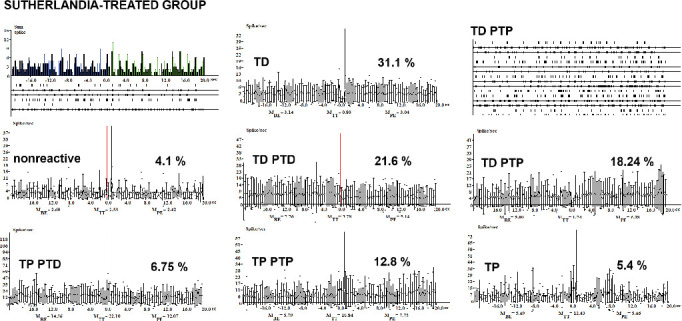
Histograms of peristimulus frequency of spike activity and the percentage distribution of indicated types of responses in hippocampal neurons in the *Sutherlandia*-treated (RSF) group following HFS of the EC are presented. Spike distribution is shown in real time for three intervals: BE, during HFS (TT), and PE. The percentage ratio of hippocampal neurons (from the total number of recorded neurons) exhibiting excitatory and inhibitory responses is also presented.

The percentage of tetanic potentiation (TP) was 8.1%. In the *Sutherlandia*-treated group (Figure 4), TD neurons comprised 31.1%, with nonreactive neurons accounting for only 4.1%. The TD-PTD percentage was 21.6%, while TD-PTP reached 18.24%. TP, TP-PTD, and TP-PTP were recorded at 5.4%, 6.75%, and 12.8%, respectively (Figure 4).

### 2.2. OFT

In addition to electrophysiological investigations, behavioral studies were carried out to identify pathological changes caused by rotenone and to assess the recovery features of *SF*.

OFTs serve as a general locomotion test and as a habit test. In PD research involving rats, various parameters are commonly assessed using OFTs to study motor deficits and nonmotor symptoms. The OFT is a widely used method to evaluate behavior and oxidative stress parameters in animal models of PD. These parameters provide valuable insights into the behavioral and physiological changes associated with PD in rat models, aiding researchers in understanding the disease mechanisms and testing potential treatments [24]. Locomotion parameters include velocity, distance traveled, signs of anxiety, and stereotypical behaviors. Changes in locomotion can reflect abnormalities in brain function and dopamine levels [25]. Reduction in exploratory activity is observed in some animal models of PD, indicating changes in behavior [24]. The reduction in exploratory activity in rats has been linked to hippocampal damage and dysfunction. Studies have shown that lesions or damage to the hippocampus can lead to memory deficits and a decrease in exploratory behavior in rats [26]. Additionally, research has highlighted the role of the hippocampus in motivated exploratory behavior, indicating that changes in hippocampal function can impact the general motor activity and exploration patterns of rats [27]. Furthermore, investigations into the effects of hippocampal lesions have demonstrated alterations in activity over time, suggesting that the hippocampus plays a crucial role in processes related to familiarization and recent memory [28].

**Figure 5:**
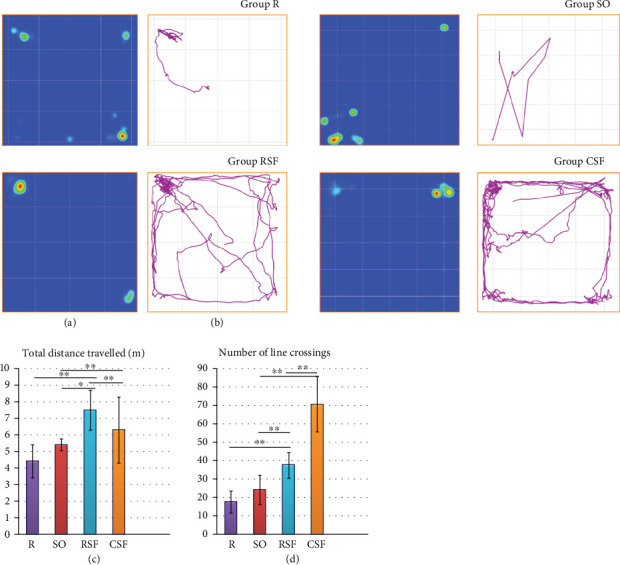
Protective activity in rats after treatments using open-field test: (a) heat map, (b, c) total distance traveled, and (d) number of crossings. Data are presented as means ± SEM. Statistical significance is indicated as ∗*p* < 0.05 and ∗∗*p* < 0.01.

In our experiments, the rats in the RSF group traveled the longest distance in the open field, followed by the CSF, SO, and R groups (Figures 5(b) and 5(c)). ANOVA revealed a significant difference in the distance traveled among the groups (*F*(3, 28) = 11.651, *p* = 0.00483). Post hoc analysis using Tukey's HSD test indicated significant differences in distance traveled between RSF and R (*p* = 0.00774), RSF and SO (*p* = 0.025), RSF and CSF (*p* = 0.00497), and CSF and SO (*p* = 0.00438). Regarding line crossings, the RSF group exhibited the highest number of crossings, followed by the CSF, SO, and R groups (Figure 5(d)). ANOVA demonstrated a significant difference in line crossings among the groups (*F*(3, 28) = 185.13, *p* < 0.001). Tukey's HSD test revealed significant differences in line crossings between RSF and R (*p* < 0.001), RSF and SO (*p* < 0.001), RSF and CSF (*p* < 0.001), and CSF and SO (*p* < 0.001) (Figure 5).

Based on the data, it can be concluded that treatment with hydroponic *SF* (RSF group) resulted in the longest distance traveled in the open field, indicating increased exploratory activity, compared to the control groups. Additionally, the RSF group showed a significant increase in line crossings, suggesting enhanced locomotor activity. These findings suggest a potential protective and stimulating effect of *SF* on exploratory and locomotor behavior in rats (Figures 5(a), 5(b), 5(c), and 5(d)).

## 3. Discussion

Animal models are essential for PD research, but no model fully replicates the human condition. Certain neurotoxins can induce PD-like symptoms in animals, including motor impairments and dopaminergic neuron loss in the substantia nigra pars compacta [29]. The pathogenesis of PD involves a complex interplay of genetic and environmental factors, including mitochondrial impairment, oxidative stress, and proteasome dysfunction. Excessive production of ROS has been implicated in the neurodegenerative process of PD, leading to the loss of dopaminergic neurons [30]. Rotenone acts as a strong inhibitor of the mitochondrial complex I, which leads to incomplete electron transfer within the mitochondrial respiratory chain, resulting in ATP depletion and oxidative stress [31, 32]. A dose of 2 mg/kg bodyweight intraperitoneally for 60 days has been shown to reliably induce PD-like features in Wistar albino rats [33, 34]. Rotenone has been implicated in the pathogenesis of PD due to its ability to cause oxidative stress and selective degeneration of nigral dopaminergic neurons, which are characteristic features of PD [35]. This model allows for testing of therapeutic interventions at clinically relevant timepoints and may improve predictive value of preclinical studies.

In our study, the CSF group displayed a 33% incidence of TD neurons (Figure 2), suggesting a suppression of synaptic transmission. The presence of TD-PTD at 22.95% and TD-PTP at 44.3% indicates complex modulation of synaptic plasticity, potentially involving both depression and potentiation mechanisms. The sunflower oil–treated group (SO, Figure 3) exhibited a significantly higher percentage of TD neurons at 93%, indicating a pronounced suppression of synaptic activity. The presence of nonreactive neurons at 7% suggests a lack of responsiveness to the applied stimuli. Treatment with rotenone (R) led to a decrease in the percentage of TD neurons to 17.74% (Figure 1), indicating a partial reversal of the synaptic suppression observed in the SO group (Figure 3). Rotenone is a widely used pesticide that causes selective degeneration of nigral dopamine neurons and PD-like symptoms in rats [36]. It is known to produce progressive neurodegeneration of dopaminergic and nondopaminergic neurons, and also of other brain cell populations such as astrocytes. Studies have shown that rotenone can induce PD pathology when brain concentrations reach 30 nM [37].

Emerging evidence indicates that hippocampal atrophy is a potential biomarker for cognitive decline in PD. Studies have shown that individuals with PD exhibit altered functional connectivity within the hippocampus, particularly decreased connectivity with regions such as the paracingulate gyrus [38]. These changes are associated with cognitive impairments and suggest that the hippocampus plays a crucial role in the pathophysiology of nonmotor symptoms [17, 38]. The presence of *α*-synuclein pathology in the hippocampus is linked to cognitive deficits observed in PD patients. Research indicates that this protein's accumulation disrupts synaptic transmission and plasticity, leading to memory impairments [16]. The hippocampus, particularly regions like the cornus ammonis (CA1-3), is critical for learning and memory, making its degeneration particularly impactful on cognitive function in PD [16]. Nonmotor symptoms are prevalent in PD, with studies reporting that up to 90% of patients experience issues such as depression, anxiety, and cognitive impairment [39]. These symptoms often precede motor dysfunction by several years and are considered core aspects of the disease's progression. The interplay between dopaminergic systems and hippocampal function is essential for understanding these manifestations [21, 38].

The presence of TP-PTD and TP-PTP at 4.8% each suggests a shift toward synaptic potentiation, potentially reflecting a recovery of synaptic function. In the RSF group (Figure 4), the percentage of TD neurons was 31.1%, comparable to that observed in the CSF group (Figure 2). The occurrence of multiple response types, including TD-PTD, TD-PTP, TP, TP-PTD, and TP-PTP, indicates a complex modulation of synaptic plasticity, likely reflecting an intricate balance between mechanisms of depression and potentiation. The presence of TD neurons in the RSF group (Figure 4) suggests a suppression of synaptic transmission similar to the CSF group. It has been shown that neurotrophic factors such as NGF, BDNF, and GDNF attenuate the selective toxicity of rotenone. The ability of these neurotrophic factors to activate the MAP kinase pathway appears to be critical for microtubule stabilization and attenuation of rotenone toxicity [36]. It was shown that *SF* influences gamma-aminobutyric acid GABA(A) synaptic transmission in the hippocampus, which could indirectly contribute to neuroprotection [40]. Additionally, ethanol extracts of *SF* have shown the ability to inhibit oxidative stress and inflammatory responses in neurons and microglial cells, suggesting a possible role in neuroprotection [36]. However, the complex modulation of synaptic plasticity observed in both groups, including the presence of both depression and potentiation mechanisms, indicates that *SF* treatment may have some impact on synaptic function. Studies have shown that *SF* has an effect on the hippocampus, particularly with regard to its interactions with the neurotransmitter GABA, which plays a crucial role in regulating excitability within neural circuits. Specifically, research reveals that *Sutherlandia* affects hippocampal glucocorticoid receptor and GABA(A)*α*1 receptor levels. Additionally, there are findings suggesting that *SF* can influence GABA(A) synaptic transmission in the hippocampus through mechanisms related to cannabinoids [40]. Furthermore, studies indicate potential anticonvulsant properties of *SF* extracts, which could be linked to these interactions with GABA signaling pathways [41].

## 4. Conclusions

While current treatments show limited efficacy in translating to clinical use in neurotoxin-induced animal models of PD, there is optimism that behavioral phenotyping in these models will pave the way for more effective treatments in the future. Our observations suggest that hydroponic *SF* could play a role in modulating hippocampal activity via GABAergic systems, highlighting its potential as a therapeutic agent in neurological disorders. Further research is warranted to elucidate the specific mechanisms involved and to explore the full therapeutic potential of hydroponic *SF* in the context of PD and other neurological conditions.

## 5. Materials and Methods

### 5.1. Ethics Statements

All animal experiments were conducted according to the principles of the National Institutes of Health (NIH) Guide for the Care and Use of Laboratory Animals and were approved by the Ethics Committee of Yerevan State Medical University, Yerevan, Armenia (ethical approval number: N4 IRB). All efforts were done to reduce animal suffering during the experimental period.

### 5.2. Animals

This study was conducted on 20 adult male albino rats, each weighing 200–240 g. The rats were housed in polycarbonate cages (five rats per cage) within a thermostatically controlled environment (temperature: 24°C; relative humidity: 45%) and maintained on a 12-h light/dark cycle. Food and water were provided *ad libitum*, and body weights were monitored regularly throughout the experimental period.

### 5.3. Chemicals and Reagents

All chemicals used in this study were purchased from Sigma-Aldrich (St. Louis, MO, USA).

### 5.4. Experimental Design

Adult male albino rats were randomly assigned to four groups and treated as follows:

1. Group CSF (control + Sutherlandia): Rats in this group received *hydroponic Sutherlandia* (82.6 mg/kg/day, oral administration) on alternate days starting from day 1 for a duration of 3 weeks.
2. Group R (rotenone): Rats were administered rotenone (2.0 mg/kg/day, subcutaneously) dissolved in sunflower oil daily for a duration of 5 weeks.
3. Group SO (sunflower oil): Rats were administered the vehicle (sunflower oil, 1 mL/kg/day, intramuscularly) daily for a period of 5 weeks.
4. Group RSF (rotenone + *Sutherlandia*): Rats in this group received rotenone (2 mg/kg/day, subcutaneously) daily for 5 weeks, followed by *hydroponic Sutherlandia* (82.6 mg/kg/day, oral administration) for 3 weeks.

All treatments were administered at doses proportional to the animals' body weights.

### 5.5. *Sutherlandia* Preparation

**Figure 6:**
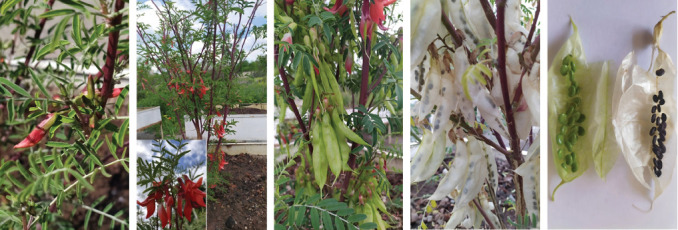
Hydroponic *Sutherlandia*: a practical method for growing *Sutherlandia* hydroponically.

*Hydroponic Sutherlandia* was purchased from the G.S. Davtyan Institute of Hydroponics Problems, NAS RA, Yerevan, Armenia (Batch No. *SF*-2023-5) (Figure 6). To prepare the aqueous extract of the plant, 82.6 mg/kg of powdered plant material was mixed with 100 mL of boiling water in a 1 L flask and allowed to brew overnight. The extract was then filtered with Whatman filter paper and freeze-dried to yield the aqueous extract, which was stored at -20°C until needed. According to previous reports, the most commonly used dose of *S. frutescens* in humans is 2.5 g dry materials (leaf) per day [42]. Given that the rat weight is 200–240 g, we calculated the rat equivalent dose for *SF*. Therefore, the rat equivalent dose for *SF* at 2.5 g per day was approximately 82.6 mg/kg per day [43].

### 5.6. In Vivo Electrophysiology

At the completion of the 3-week period for the CSF group, the 5-week period for the R and SO-treated groups, and the 8-week period for the RSF group, the animals were anesthetized with urethane (1.1 g/kg, intraperitoneally (ip), immobilized with 1% ditiline (25 mg/kg, ip), positioned in a stereotaxic frame, and placed on artificial ventilation. Anesthetized and shaved rats were placed in a stereotactic frame, and artificial ventilation was used. The sample of “encephaleisolé” rat was obtained by transection of the spinal cord (T2–T3) using ultrasound. Extracellular spike activity from hippocampal neurons was recorded using a microelectrode (tip diameter 1–2 *μ*m; resistance 1.5–2.5 M*Ω*) filled with a 3 M KCl solution. The microelectrode was incrementally advanced into the hippocampus following stereotaxic coordinates based on the rat brain atlas (AP −3.2 to −3.5; L ±1.5 to −3.5; DV +2.8 to +4.0 mm) [44]. HFS (100 Hz for 1 s) of the ipsilateral EC was delivered through bipolar silver electrodes. Stimuli were applied using rectangular current pulses (duration: 0.05 ms; amplitude: 0.6–0.8 mA). The stimulating electrode was positioned in the ipsilateral EC according to stereotaxic coordinates (AP −9.0; L ±3.5; DV +4.0 mm). The heterogeneity of interspike intervals (or spike frequency) in the pre- and poststimulus impulse flow was statistically analyzed using the Student's *t*-test and the Mann–Whitney *U* test, as described in our previous studies [45, 46].

### 5.7. Behavioral Studies

#### 5.7.1. OFT

The OFT is a widely used behavioral assessment tool to evaluate spontaneous exploratory activity and anxiety-related behaviors in animals, including rodents. In this test, the apparatus typically consists of a square or rectangular arena with black or white walls and floor, with dimensions such as 60 × 60 cm (as mentioned in the question) and walls that are 40 cm in height [47]. The black melamine material for the walls and floor is commonly used to provide a homogeneous and uniform background, which helps to minimize potential confounding factors such as visual cues. The OFT is a noninvasive and relatively simple test that allows researchers to assess locomotor activity, anxiety, and other behaviors in animals, providing valuable information for studying various neurological disorders, including PD. In this test, rats were placed in a square without a ceiling, typically for 5 min, and their behavior was recorded and analyzed [48]. To evaluate the ability of the rats to adapt to a new environment and the detective activities, the time and distance spent in the central area were traced and recorded. These parameters are considered indices of locomotor activity. After each test, 75% ethyl alcohol was used to clean the urine and feces left behind by the other rats which could produce odor signals that would interfere the next rats to be tested. The following dependent variables were recorded: total distance moved and number of line crossings [49, 50].

### 5.8. Statistical Analyses

For electrophysiological data analysis, we used *t*-criteria of Student's *t*-test and the reliability of differences of interspike intervals before, after, and during HFS. All *p* values were determined by a two-sample unpaired Student's *t*-test. The spread of the data where indicated is the SD of the mean. To increase reliability of statistical evaluations, we also used the nonparametric method of verification by application of Wilcoxon two-sample test. Significant differences between the obtained values were analyzed using GraphPad Prism software (Version 5.0) using one-way ANOVA, followed by Tukey's post hoc test.

## Data Availability

The data that support the findings of this study are available from the corresponding author upon reasonable request.

## References

[1] von Wrangel C., Schwabe K., John N., Krauss J. K., Alam M. (2015). The rotenone-induced rat model of Parkinson's disease: behavioral and electrophysiological findings. *Behavioural Brain Research*.

[2] Sia C. (2004). Spotlight on ethnomedicine: usability of *Sutherlandia frutescens* in the treatment of diabetes. *The Review of Diabetic Studies*.

[3] Chadwick W. A., Roux S., van de Venter M., Louw J., Oelofsen W. (2007). Anti-diabetic effects of *Sutherlandia frutescens* in Wistar rats fed a diabetogenic diet. *Journal of Ethnopharmacology*.

[4] Skerman N. B., Joubert A. M., Cronjé M. J. (2011). The apoptosis inducing effects of Sutherlandia spp. extracts on an oesophageal cancer cell line. *Journal of Ethnopharmacology*.

[5] Shaik S., Singh N., Nicholas A. (2011). Comparison of the selected secondary metabolite content present in the cancer-bush *Lessertia (Sutherlandia) frutescens* L. extracts. *African Journal of Traditional, Complementary, and Alternative Medicines*.

[6] Jiang J., Chuang D. Y., Zong Y. (2014). Sutherlandia frutescens ethanol extracts inhibit oxidative stress and inflammatory responses in neurons and microglial cells. *PLoS One*.

[7] Sharifi-Rad M., Lankatillake C., Dias D. A. (2020). Impact of natural compounds on neurodegenerative disorders: from preclinical to pharmacotherapeutics. *Journal of Clinical Medicine*.

[8] Lei W., Browning J. D., Eichen P. A. (2015). Unveiling the anti-inflammatory activity of Sutherlandia frutescens using murine macrophages. *International Immunopharmacology*.

[9] Chuang D. Y., Cui J., Simonyi A. (2014). Dietary Sutherlandia and elderberry mitigate cerebral ischemia-induced neuronal damage and attenuate p47phox and phospho-ERK1/2 expression in microglial cells. *ASN Neuro*.

[10] Folk W. R., Smith A., Song H. (2016). Does concurrent use of some botanicals interfere with treatment of tuberculosis?. *Neuromolecular Medicine*.

[11] Coleman C., Martin I. (2022). Unraveling Parkinson's disease neurodegeneration: does aging hold the clues?. *Journal of Parkinson's Disease*.

[12] Chen R., Berardelli A., Bhattacharya A. (2022). Clinical neurophysiology of Parkinson's disease and parkinsonism. *Clinical Neurophysiology Practice*.

[13] Van Laar A. D., Webb K. R., Keeney M. T. (2023). Transient exposure to rotenone causes degeneration and progressive parkinsonian motor deficits, neuroinflammation, and synucleinopathy. *NPJ Parkinson's Disease*.

[14] Jellinger K. A. (2024). Pathobiology of cognitive impairment in parkinson disease: challenges and outlooks. *International Journal of Molecular Sciences*.

[15] Camicioli R., Moore M. M., Kinney A., Corbridge E., Glassberg K., Kaye J. A. (2003). Parkinson's disease is associated with hippocampal atrophy. *Movement Disorders*.

[16] Villar-Conde S., Astillero-Lopez V., Gonzalez-Rodriguez M. (2021). The human hippocampus in Parkinson's disease: an integrative stereological and proteomic study. *Journal of Parkinson's Disease*.

[17] Llewelyn L. E., Kornisch M., Park H., Ikuta T. (2022). Hippocampal functional connectivity in Parkinson’s disease. *Neurodegenerative Diseases*.

[18] Kumaresan M., Khan S. (2021). Spectrum of non-motor symptoms in Parkinson's disease. *Cureus*.

[19] Titova N., Chaudhuri K. R. (2018). Non-motor Parkinson disease: new concepts and personalised management. *The Medical Journal of Australia*.

[20] Kalia L. V., Lang A. E. (2015). Parkinson's disease. *Lancet*.

[21] Radad K., Moldzio R., Krewenka C., Kranner B., Rausch W. D. (2023). Pathophysiology of non-motor signs in Parkinson’s disease: some recent updating with brief presentation. *Exploration of Neuroprotective Therapy*.

[22] Poewe W. (2008). Non-motor symptoms in Parkinson's disease. *European Journal of Neurology*.

[23] Li X., Chen C., Pan T. (2024). Trends and hotspots in non-motor symptoms of Parkinson's disease: a 10-year bibliometric analysis. *Frontiers in Aging Neuroscience*.

[24] Campos F. L., Carvalho M. M., Cristovão A. C. (2013). Rodent models of Parkinson's disease: beyond the motor symptomatology. *Frontiers in Behavioral Neuroscience*.

[25] Tuon T., Meirelles S. S., de Moura A. B. (2021). Behavior and oxidative stress parameters in rats subjected to the animal's models induced by chronic mild stress and 6-hydroxydopamine. *Behavioural Brain Research*.

[26] Mumby D. G., Gaskin S., Glenn M. J., Schramek T. E., Lehmann H. (2002). Hippocampal damage and exploratory preferences in rats: memory for objects, places, and contexts. *Learning & Memory*.

[27] Alvarez E. O., Alvarez P. A. (2008). Motivated exploratory behaviour in the rat: the role of hippocampus and the histaminergic neurotransmission. *Behavioural Brain Research*.

[28] Roberts W. W., Dember W. N., Brodwick M. (1962). Alternation and exploration in rats with hippocampal lesions. *Journal of Comparative and Physiological Psychology*.

[29] Lama J., Buhidma Y., Fletcher E. J. R., Duty S. (2021). Animal models of Parkinson's disease: a guide to selecting the optimal model for your research. *Signals*.

[30] Stoker T. B., Greenland J. C. (2018). *Parkinson’s disease: Pathogenesis and clinical aspects*.

[31] Eriksen J. L., Wszolek Z., Petrucelli L. (2005). Molecular pathogenesis of Parkinson disease. *Archives of Neurology*.

[32] Wood D. M., Alsahaf H., Streete P., Dargan P. I., Jones A. L. (2005). Fatality after deliberate ingestion of the pesticide rotenone: a case report. *Critical Care*.

[33] Alam M., Schmidt W. J. (2002). Rotenone destroys dopaminergic neurons and induces parkinsonian symptoms in rats. *Behavioural Brain Research*.

[34] Duty S., Jenner P. (2011). Animal models of Parkinson's disease: a source of novel treatments and clues to the cause of the disease. *British Journal of Pharmacology*.

[35] Heinz S., Freyberger A., Lawrenz B., Schladt L., Schmuck G., Ellinger-Ziegelbauer H. (2017). Mechanistic investigations of the mitochondrial complex I inhibitor rotenone in the context of pharmacological and safety evaluation. *Scientific Reports*.

[36] Jiang Q., Yan Z., Feng J. (2006). Neurotrophic factors stabilize microtubules and protect against rotenone toxicity on dopaminergic neurons. *The Journal of Biological Chemistry*.

[37] Pamies D., Block K., Lau P. (2018). Rotenone exerts developmental neurotoxicity in a human brain spheroid model. *Toxicology and Applied Pharmacology*.

[38] Calabresi P., Castrioto A., Di Filippo M., Picconi B. (2013). New experimental and clinical links between the hippocampus and the dopaminergic system in Parkinson's disease. *Lancet Neurology*.

[39] Frucht S. J. (2004). Parkinson disease: an update. *Neurologist*.

[40] Smith C., van Vuuren M. J. (2014). Central and peripheral effects of *Sutherlandia frutescens* on the response to acute psychological stress. *Experimental Biology and Medicine*.

[41] Ojewole J. A. (2008). Anticonvulsant property of Sutherlandia frutescens R. BR. (variety Incana E. MEY.) (Fabaceae) shoot aqueous extract. *Brain Research Bulletin*.

[42] Mills E., Cooper C., Seely D., Kanfer I. (2005). African herbal medicines in the treatment of HIV: Hypoxis and Sutherlandia. An overview of evidence and pharmacology. *Nutrition Journal*.

[43] Lei W., Browning J. D., Eichen P. A. (2016). An investigation into the immunomodulatory activities of Sutherlandia frutescens in healthy mice. *PLoS One*.

[44] Paxinos G., Watson C. (2005). *The rat brain in stereotaxic coordinates: compact*.

[45] Darbinyan L. V., Hambardzumyan L. E., Simonyan K. V., Chavushyan V. A., Manukyan L. P., Sarkisian V. H. (2017). Rotenone impairs hippocampal neuronal activity in a rat model of Parkinson's disease. *Pathophysiology*.

[46] Darbinyan L. V., Hambardzumyan L. E., Simonyan K. V. (2017). Protective effects of curcumin against rotenone-induced rat model of Parkinson's disease: in vivo electrophysiological and behavioral study. *Metabolic Brain Disease*.

[47] La-Vu M., Tobias B. C., Schuette P. J., Adhikari A. (2020). To approach or avoid: an introductory overview of the study of anxiety using rodent assays. *Frontiers in Behavioral Neuroscience*.

[48] Jantzie L. L., Oppong A. Y., Conteh F. S. (2018). Repetitive neonatal erythropoietin and melatonin combinatorial treatment provides sustained repair of functional deficits in a rat model of cerebral palsy. *Frontiers in Neurology*.

[49] Prut L., Belzung C. (2003). The open field as a paradigm to measure the effects of drugs on anxiety-like behaviors: a review. *European Journal of Pharmacology*.

[50] Kuniishi H., Ichisaka S., Yamamoto M. (2017). Early deprivation increases high-leaning behavior, a novel anxiety-like behavior, in the open field test in rats. *Neuroscience Research*.

